# Circulating adipsin is associated with asymptomatic carotid atherosclerosis in obese adults

**DOI:** 10.1186/s12872-021-02329-3

**Published:** 2021-10-25

**Authors:** Jinhua Zhang, Fei Teng, Lingling Pan, Dan Guo, Jianfang Liu, Kangli Li, Youwen Yuan, Wenyuan Li, Huijie Zhang

**Affiliations:** 1 Key Laboratory of Functional and Clinical Translational Medicine, Department of General Medicine, Xiamen Medical College, Xiamen, China; 2 Department of Endocrinology and Metabolism, Nanfang Hospital, Southern Medical University, 1838 North Guangzhou Road, Guangzhou, 510515 China; 3 Department of Endocrinology and Metabolism, Tongji Hospital, Tongji University, Shanghai, China; 4 The First Affiliated Hospital of Xiamen University, Xiamen, China; 5 Department of Medical Imaging Center, Nanfang Hospital, Southern Medical University, Guangzhou, China

**Keywords:** Adipsin, Obesity, Carotid intima-media thickness, Cardiovascular disease, Asymptomatic carotid atherosclerosis

## Abstract

**Background:**

Adipsin has been identified as a secreted adipokine that plays a critical pathogenic role in metabolic disorders. However, it is not clear regarding the association of circulating adipsin with cardiovascular disease (CVD). This study will explore the association between circulating adipsin and asymptomatic carotid atherosclerosis in Chinese obese adults.

**Methods:**

A total of 483 obese adult subjects (aged 40 years or older) were enrolled in this study. Serum adipsin concentrations and carotid intima-media thickness (CIMT) were measured to determine these associations.

**Results:**

Individuals with increased CIMT and asymptomatic carotid atherosclerosis had lower levels of circulating adipsin than controls (both *p* < 0.05). The prevalence of asymptomatic carotid atherosclerosis was significantly higher in subjects with lower levels of serum adipsin than those with higher values (42.5% vs. 36.7%, *p* < 0.05). Notably, subjects in the lowest quartile of serum adipsin were 1.94 times (*p* = 0.059) more likely to have increased CIMT and 2.91 times (*p* = 0.03) more likely to have asymptomatic carotid atherosclerosis than those in the highest quartile in multivariable logistic regression analyses, adjusting for age, gender, current smoking, alcohol consumption, physical activity, BMI, systolic BP, fasting glucose, total cholesterol, HDL-c, and HOMA-IR. However, such associations with circulating adipsin were not noted for atherosclerotic plaque.

**Conclusions:**

These findings suggest that circulating adipsin concentrations are a potential marker of risks of increased CIMT and asymptomatic carotid atherosclerosis in obese Chinese adults.

## Introduction

Cardiovascular disease (CVD) is the leading cause of morbidity and mortality worldwide [1], which gained worldwide attention. It includes myocardial infarction, hypertension, atherosclerosis, vascular disease, and stroke [2], affecting approximately 290 million people in China [3, 4]. However, almost half of patients who died from CVD can have no clinical symptoms of CVD [5]. Subclinical CVD is a broad term that includes carotid atherosclerosis and arterial stiffness strongly associated with future CVD events [6, 7]. Asymptomatic carotid atherosclerosis is the most common type of subclinical CVD [8]. Identification of risk factors for the development of symptomatic carotid atherosclerosis could prevent and delay the progress of CVD. Numerous risk factors have been well established, including age, smoking, physical inactivity, obesity, hypertension, diabetes, and hyperlipidemia [9, 10]. Excess adiposity is strongly associated with the risk of CVD in the general population [11].

It has been proposed that obesity-associated insulin resistance and low-grade inflammation may largely account for the development of CVD [12, 13]. However, the mechanisms by which insulin resistance and obesity promote CVD risk remain uncertain. Adipose tissue has been identified as an energy storage organ. Meanwhile, it also plays as an essential endocrine organ secreting adipokines [14, 15]. Several adipokines, including Leptin, fetuin-A, and adiponectin, are associated with risk of obesity, type 2 diabetes (T2DM), metabolic syndrome (MetS), and CVD [13, 16–20].

Adipsin, a member of the serine protease gene family, is recently identified as a novel secreted adipokine, playing an important role in modulating systemic energy metabolism [21]. Previous studies have shown that adipsin plays a significant role in controlling blood glucose levels and insulin sensitivity [22–24]. Banoy et al. reported that adipsin plays a pivotal role in preserving beta cells and protecting from type 2 diabetes in diabetic mice and humans through splitting complement factor B in the alternative complement pathway and catalyzing the formation of complement component C3a [23]. Levels of adipsin are decreased in patients with T2DM [25]. As described above, the associations between adipsin and metabolic disorders have been previously discussed. Given the connection between obesity-associated metabolic disorder and the risk of CVD, circulating adipsin might be a candidate marker of CVD risk. However, there is little evidence regarding the association between circulating adipsin and CVD in the community cohort study. In this study, we aimed to explore the association between circulating adipsin and the risk of asymptomatic carotid atherosclerosis in Chinese obese adults.

## Methods

### Study participants

Between April 2011 and December 2013, 1536 obese subjects aged 40 years or older were recruited from the community in China after physical examination [26]. Obesity was defined as an elevated waist circumference (> 90 cm in males or 80 cm in females). Subjects with the following conditions were excluded from this study: acute or chronic viral hepatitis, drug-induced liver diseases, obstructive biliary diseases, Wilson's disease, auto-immune disease, total parenteral nutrition, cancer, or known hypothyroidism or hyperthyroidism. A total of 520 participants were randomly chosen to receive carotid ultrasonography for the measurement of carotid intima-media thickness. Participants with missing data for serum adipsin levels were further excluded in this study. In total, our final analysis included 483 participants.

All subjects provided written informed consent. The study protocol was approved by the Institutional Review Board of the First Affiliated Hospital of Xiamen University and Nanfang Hospital of Southern Medical University. The methods were carried out in accordance with the approved guidelines.

### Clinical and biochemical measurements

A spring scale and a vertical ruler measure were used to obtain bodyweight and height measurements, respectively. Body mass index (BMI) measures total body adiposity. Waist circumference was measured at the level of the umbilicus. Blood pressure (BP) was assessed in triplicate using an electronic sphygmomanometer (OMRON Company). Body fat mass was determined using the HOLOGIC whole body DXA system (Hologic Inc., Bedford, MA). Each measurement was performed at least three times, and the mean value was used for analysis.

Each subject underwent 75-g oral glucose tolerance tests and blood biochemical measurements a 12 h fast. Plasma glucose, high-density lipoprotein cholesterol (HDL-c), triglycerides (TG), and total cholesterol (TC) were measured by enzymatic colorimetric methods with a Hitachi 7450 analyzer (Hitachi, Tokyo, Japan). Low-density lipoprotein cholesterol (LDL-C) was calculated by Friedewald’s formula. Fasting plasma glucose concentrations and 2-h glucose concentrations were measured using the glucose oxidase method. Serum insulin concentrations were measured using electrochemiluminescence immunoassay (Roche Elecsys Insulin Test, Roche Diagnostics, Mannheim, Germany). Insulin resistance was estimated using HOMA of insulin resistance (HOMA-IR).

### Measurement of carotid intima-media thickness

The Carotid intima-media thickness (CIMT) was performed and analyzed by a single trained sonographer using a high-resolution B-mode tomographic ultrasound system (Philips-ATL HDI-5000, Philips Medical Systems, Bothell, WA, USA) [27]. The far wall of both left and right common carotid arteries at 1.0 cm proximal to the bifurcation were scanned. The mean of the maximum CIMT reading of right and left far walls for common, bulb, and internal segments were calculated [27]. Plaque was defined as an echogenic focal structure encroaching the vessel lumen with at least 50% greater thickness than that found in surrounding areas. All these measurements were repeated three times.

### Serum Adipsin measurement

Serum adipsin concentrations were measured by using enzyme-linked immunosorbent assay (ELISA) kits (AssayPro, St. Charles, MO, USA) according to the manufacturer’s instructions. The assay has been shown to be highly sensitive to human adipsin with a sensitivity of 0.0021 ug/ml. The linear range of the standard was 0.001875–0.120 ug/ml, and the intra and inter assay variations were both less than 10%.

### Statistical analysis

Data are presented as means ± standard deviation (S.D) for normally distributed variables, median (interquartile range) for variables that were not normally distributed, and numbers (percentages) for the categorical variables. Data that were not normally distributed were logarithmically transformed before analysis. The subjects were classified into four quartiles according to their serum level of adipsin.

Chi-square test or logistic regression models were used to examine differences in categorical variables in different study groups. Analyses of covariance were performed using general linear models (GLM) to test differences in study variables between different quartiles of serum adipsin. The Benjamini–Hochberg method was used to correct the p-values for the multiple comparisons. Multivariable logistic regression models were used to examine the association of serum adipsin levels with risks of increased CIMT, adjusted for age, gender, smoking, alcohol consumption, physical activity, BMI, systolic BP, glucose, triglyceride, HDL-c, HOMA-IR, and body fat mass. Increased CIMT was defined as the average CIMT ≥ 0.8 mm [28]. Asymptomatic carotid atherosclerosis was defined as increased CIMT or the presence of atherosclerotic plaque. Two-sided values of *p* < 0.05 were considered statistically significant. All statistical analyses were performed with SAS version 9.3 (SAS Institute, Cary, NC).

## Results

The demographic, clinical, and laboratory characteristics were shown in Table 1. Obese subjects with increased CIMT had higher levels of current smokers, systolic blood pressure, total cholesterol, LDL-c, fasting glucose, and visceral fat compared with those without increased CIMT (all *p* < 0.05). Of interest, subjects with increased CIMT had lower serum adipsin levels than subjects without increased CIMT (25.7 ± 7.1 ng/ml vs. 27.4 ± 10.7 ng/ml, *p* = 0.002). Besides, patients with atherosclerotic plaques had higher total cholesterol and LDL-c than subjects without atherosclerotic plaque (*p* < 0.05). There are no differences in serum adipsin in subjects without atherosclerotic plaque compared to subjects with atherosclerotic plaque. Additionally, subjects with asymptomatic carotid atherosclerosis had an unfavorable metabolic profile, including higher levels of total cholesterol, LDL-c, and visceral fat. Importantly, subjects with asymptomatic carotid atherosclerosis had lower serum adipsin levels than subjects without asymptomatic carotid atherosclerosis (26.4 ± 9.4 ug/ml vs. 27.2 ± 10.2 ug/ml, *p* = 0.004).

**Table 1:** Clinical and biochemical characteristics of obese subjects

Variables	Increased CIMT			Atherosclerotic plaque			Asymptomatic carotid atherosclerosis*		
	Yes	No	P-value^§^	Yes	No	P-value^§^	Yes	No	P-value^§^
Sample size	138	345		116	367		209	274	
Age (years)	56.0 ± 6.2	53.4 ± 7.4	< 0.001	58.1 ± 6.1	52.9 ± 7.0	< 0.001	56.7 ± 6.3	52.2 ± 7.2	< 0.001
Gender (male n, %)	56(40.6)	74(21.5)	< 0.001	41(35.3)	89(24.3)	0.019	75(35.9)	55(20.1)	< 0.001
BMI (kg/m^2^)	27.6 ± 2.9	27.1 ± 2.9	0.081	26.9 ± 3.0	27.3 ± 2.9	0.225	27.3 ± 3.0	27.2 ± 2.9	0.599
Waist circumference (cm)	95.1 ± 7.1	93.9 ± 6.8	0.755	94.0 ± 6.5	94.3 ± 7.0	0.079	94.6 ± 6.9	93.9 ± 6.9	0.550
Current smokers (n, %)	31(22.5)	43(12.5)	0.006	20(17.2)	54(14.7)	0.511	41(19.6)	33(12.0)	0.022
Systolic BP (mmHg)	134.8 ± 17.5	127.4 ± 15.2	0.001	133.7 ± 18.5	128.2 ± 15.2	0.148	132.7 ± 17.6	127.1 ± 14.7	0.084
Diastolic BP (mmHg)	80.0 ± 10.5	77.1 ± 9.8	0.052	78.7 ± 10.6	77.7 ± 9.9	0.733	78.7 ± 10.4	77.3 ± 9.8	0.594
Triglycerides (mmol/L)	1.83 (1.31–2.35)	1.51 (1.13–2.12)	0.682	1.65 (1.19–2.14)	1.58 (1.17–2.20)	0.780	1.70 (1.20–2.23)	1.54 (1.12–2.14)	0.591
Total cholesterol (mmol/L)	6.07 ± 1.07	5.64 ± 0.94	< 0.001	6.03 ± 1.10	5.67 ± 0.95	0.014	5.96 ± 1.04	5.61 ± 0.94	0.003
LDL-cholesterol (mmol/L)	4.00 ± 1.00	3.68 ± 0.96	0.009	4.06 ± 1.10	3.68 ± 0.92	0.014	3.96 ± 0.99	3.63 ± 0.94	0.012
HDL-cholesterol (mmol/L)	1.28 ± 0.22	1.32 ± 0.27	0.542	1.30 ± 0.24	1.31 ± 0.26	0.687	1.28 ± 0.23	1.33 ± 0.27	0.204
Fasting glucose (mmol/L)	5.75 ± 0.91	5.53 ± 0.70	0.032	5.65 ± 1.01	5.57 ± 0.68	0.911	5.65 ± 0.82	5.55 ± 0.74	0.789
2-h glucose (mmol/L)	8.07 ± 2.34	7.95 ± 2.26	0.734	8.05 ± 2.59	7.97 ± 2.18	0.702	7.97 ± 2.29	8.00 ± 2.28	0.406
HOMA-IR	2.91 (2.26–3.87)	2.83 (1.84–4.08)	0.147	2.62 (1.90–4.07)	2.97 (1.95–4.01)	0.736	2.72 (1.97–3.86)	2.98 (1.93–4.11)	0.965
Serum adipsin (ug/ml)	5.13 ± 1.43	5.47 ± 2.14	0.002	5.50 ± 2.20	5.33 ± 1.89	0.475	5.28 ± 1.88	5.44 ± 2.04	0.004
Body fat mass(kg)	22.9 ± 5.2	23.2 ± 4.5	0.432	22.3 ± 4.7	23.4 ± 4.7	0.228	5.29 ± 1.88	5.44 ± 2.04	0.876
Visceral fat (cm^2^)	144.3 ± 45.7	115.2 ± 40.1	< 0.001	127.6 ± 42.5	122.3 ± 44.1	0.159	134.2 ± 44.8	115.4 ± 41.2	0.036

^*^Asymptomatic carotid atherosclerosis is defined as increased CIMT or presence of atherosclerotic plaque

CIMT = Carotid intima-media thickness; BMI = body mass index; HOMA-IR = homeostasis model assessment of insulin resistance;

Data are presented as the mean ± SD or median (interquartile range) or numbers (percentages)

^§^Adjusted for age and gender

The clinical and biochemical characteristics by quartiles of serum adipsin levels were shown in Table 2. systolic BP, diastolic BP, total cholesterol, triglyceride, LDL-c, HDL-c, fasting glucose, and visceral fat showed no significant differences among the four quartiles of serum adipsin levels, adjusted with age and gender. Compared to subjects in the lowest quartile of serum adipsin levels, those in the highest quartile had significantly higher levels of BMI, waist circumference, body fat mass, and lower levels of postprandial glucose and HOMA-IR (both p < 0.05). Additionally, CIMT was reduced gradually with the increase of serum adipsin (*p* = 0.009). Interestingly, the prevalence of asymptomatic carotid atherosclerosis was significantly higher in subjects with lower levels of serum adipsin than those with higher values (42.5% vs. 36.7%, *p* < 0.05). However, the prevalence of atherosclerotic plaque showed no significant difference across quartiles of serum adipsin levels.

**Table 2:** Clinical and biochemical characteristics by quartiles of serum adipsin levels

Variables	Serum adipsin level				P-value for trend^§^:
	Q1	Q2	Q3	Q4	
Sample size	120	121	122	120	
Serum adipsin (ug/ml)	3.52 ± 0.61	4.53 ± 0.35	5.49 ± 0.44	7.96 ± 2.01	< 0.001
Age (years)	53.0 ± 7.6	53.5 ± 7.0	54.0 ± 7.2	56.0 ± 6.5‡	0.007
Gender (male n, %)	32 (26.7)	33 (27.3)	33 (27.1)	32 (26.7)	0.990
BMI (kg/m^2^)	26.7 ± 2.8	27.1 ± 2.8	27.6 ± 3.0	27.6 ± 3.1†	0.036
Waist circumference (cm)	92.8 ± 6.6	93.6 ± 6.9	94.8 ± 6.9†	95.7 ± 6.9‡	0.007
Current smokers (n, %)	17(14.2)	16(13.2)	23(18.9)	18(15.0)	0.633
Systolic BP (mmHg)	128.1 ± 15.3	129.6 ± 17.3	128.8 ± 13.7	131.6 ± 18.3	0.705
Diastolic BP (mmHg)	77.2 ± 9.6	78.0 ± 10.9	78.0 ± 9.6	78.4 ± 10.3	0.821
Triglycerides (mmol/L)	1.50 (1.13–2.14)	1.57 (1.24–2.23)	1.60 (1.14–2.16)	1.70 (1.22–2.25)	0.268
Total cholesterol (mmol/L)	5.67 ± 1.06	5.71 ± 0.94	5.81 ± 0.99	5.84 ± 0.99	0.737
LDL- cholesterol (mmol/L)	3.66 ± 1.08	3.67 ± 0.92	3.89 ± 0.99	3.86 ± 0.90	0.248
HDL-cholesterol (mmol/L)	1.32 ± 0.26	1.29 ± 0.28	1.32 ± 0.23	1.31 ± 0.26	0.644
Fasting glucose (mmol/L)	5.64 ± 0.89	5.67 ± 1.03	5.50 ± 0.50	5.55 ± 0.53	0.183
2-h glucose (mmol/L)	8.37 ± 2.55	8.12 ± 2.49	7.63 ± 1.80†	7.83 ± 2.17†	0.027
HOMA-IR	2.97 (1.82- 4.06)	3.10 (2.14- 4.52)	2.58 (1.78- 3.64)	2.80 (2.08- 4.00)	0.015
Body fat mass(kg)	22.2 ± 4.7	22.9 ± 4.3	23.6 ± 4.8†	23.9 ± 4.9‡	0.017
Visceral fat (cm^2^)	116.3 ± 38.8	126.6 ± 50.7	124.0 ± 44.9	127.4 ± 39.0	0.302
CIMT (mm):	0.74 ± 0.16	0.76 ± 0.17	0.73 ± 0.15	0.71 ± 0.13†	0.009
Atherosclerotic plaque (n, %)	31 (25.8)	28 (23.1)	25 (20.5)	32 (26.7)	0.259
Asymptomatic carotid atherosclerosis*	51 (42.5)	60 (50.0)	54 (44.3)	44 (36.7)	0.017

^*^Asymptomatic carotid atherosclerosis is defined as increased CIMT or presence of atherosclerotic plaque

CIMT = Carotid intima-media thickness; BMI = body mass index; HOMA-IR = homeostasis model assessment of insulin resistance;

Data are presented as the mean ± SD or median (interquartile range) or numbers (percentages)

^§^Adjusted for age and gender

^†^Adjusted P < 0.05compared with Q1 of serum adipsin

^‡^Adjusted P < 0.01compared with Q1 of serum adipsin

**Fig. 1:**
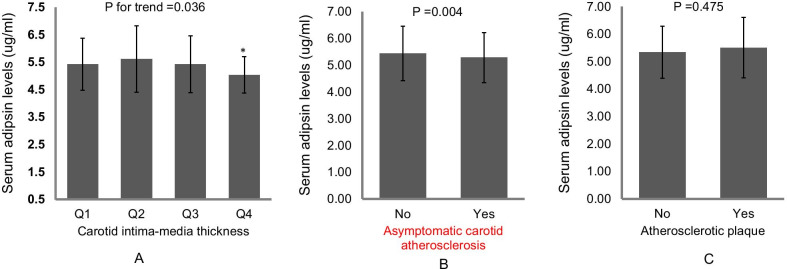
Serum adipsin levels in asymptomatic carotid atherosclerosis and normal subjects. **a** Carotid intima-media thickness according to quartiles of serum adipsin levels. **b** Relationship between serum adipsin levels and asymptomatic carotid atherosclerosis. **c** Relationship between serum adipsin levels and atherosclerotic plaque

As shown in Fig. 1, CIMT was reduced gradually with the increase of serum adipsin in obese subjects (*p* = 0.036). Serum adipsin was significantly lower in patients with subclinical cardiovascular disease than non-subclinical cardiovascular disease subjects (*p* = 0.004). However, compared with subjects without atherosclerotic plaques, there was no significant difference between the two groups.

Multivariable logistic regression was utilized to estimate adjusted odds ratios (ORs) for the association of serum adipsin levels with increased CIMT, atherosclerotic plaque, and asymptomatic carotid atherosclerosis are shown in Table 3. The risk of increased CIMT was positively associated with HOMA-IR (*p* = 0.043) and visceral fat (*p* < 0.001). Meanwhile, serum adipsin showed a significant association with increased CIMT after adjustment for age and gender (*p* = 0.003). Importantly, subjects in the second quartile and the third quartile of serum adipsin were 3.536 times (p = 0.001) and 2.443 times (p = 0.006) more likely to have increased CIMT than those in the highest quartile. Furthermore, the risk of increased CIMT was still significantly higher in subjects in the lower quartile of serum adipsin than those in the highest quartile, even after adjusting for age, gender, current smoking, alcohol consumption, and physical activity. In addition, this relationship remained significant after further adjusting for age, gender, current smoking, alcohol consumption, physical activity, systolic BP, glucose, total cholesterol, triglyceride, and HDL-c. The risks of asymptomatic carotid atherosclerosis were reduced by 29.6% per 1 SD increase in serum adipsin levels (log-transformed). Notably, the ORs for asymptomatic carotid atherosclerosis remained significant OR [(95% CI) 0.705 (0.567–0.876), p = 0.002], even after adjusting for age, sex, current smoking, alcohol consumption, and physical activity. After further adjustment for systolic BP, glucose, total cholesterol, triglyceride, and HDL-c, the relationship between serum adipsin and asymptomatic carotid atherosclerosis remained significant [OR (95% CI) 0.665 (0.532–0.832), p < 0.001]. Similarly, subjects in the lower quartiles also showed a significantly elevated risk for asymptomatic carotid atherosclerosis compared to those in the fourth quartile (all *p* < 0.05). However, such associations with circulating adipsin were not noted for atherosclerotic plaque.

**Table 3:** Adjusted odds ratios (ORs) with associated 95% confidence interval (CI) for asymptomatic carotid atherosclerosis

Variables	Increased CIMT			Atherosclerotic plaque			Asymptomatic carotid atherosclerosis*		
	OR	95% CI	P-value	OR	95% CI	P-value	OR	95% CI	P-value
Model 1									
BMI (kg/m^2^)	1.244	0.992–1.560	0.059	0.789	0.606–1.026	0.077	1.048	0.846–1.299	0.669
Waist circumference (cm)	1.037	0.827–1.300	0.754	0.752	0.577–0.979	0.034	0.932	0.752–1.156	0.523
HOMA-IR	1.491	1.013–2.192	0.043	1.040	0.685–1.579	0.854	1.098	0.764–1.577	0.613
Body fat mass (Kg)	1.086	0.864–1.365	0.482	0.797	0.613–1.035	0.089	0.991	0.798–1.231	0.937
Visceral fat (cm^2^)	1.736	1.374–2.194	< 0.001	0.853	0.666–1.092	0.206	1.271	1.022–1.581	0.031
Serum adipsin (ug/ml)	0.691	0.541–0.882	0.003	0.877	0.696–1.107	0.269	0.704	0.567–0.875	0.002
Serum adipsin (ug/ml)									
(Quartile 1 vs. Quartile 4)	1.762	0.916–3.390	0.090	1.435	0.764–2.696	0.261	2.002	1.120–3.579	0.019
(Quartile 2 vs. Quartile 4)	3.536	1.898–6.588	< 0.001	1.144	0.608–2.152	0.677	2.528	1.431–4.469	0.001
(Quartile 3 vs. Quartile 4)	2.443	1.299–4.595	0.006	0.839	0.437–1.609	0.597	1.813	1.022–3.216	0.042
Model 2									
BMI (kg/m^2^)	1.251	0.996- 1.570	0.054	0.784	0.602–1.021	0.071	1.049	0.846–1.301	0.660
Waist circumference (cm)	1.043	0.831–1.310	0.714	0.759	0.582–0.990	0.042	0.936	0.754–1.162	0.551
HOMA-IR	1.501	1.015–2.218	0.042	1.011	0.806–1.269	0.922	1.095	0.761–1.577	0.625
Body fat mass (Kg)	1.095	0.871–1.378	0.437	0.801	0.616–1.041	0.097	0.998	0.803–1.240	0.983
Visceral fat (cm^2^)	1.777	1.400–2.255	< 0.001	0.850	0.662–1.092	0.205	1.284	1.030–1.600	0.026
Serum adipsin (ug/ml)	0.690	0.540–0.883	0.003	0.879	0.697–1.109	0.277	0.705	0.567–0.876	0.002
Serum adipsin (ug/ml)									
(Quartile 1 vs. Quartile 4)	1.788	0.930–3.441	0.082	1.426	0.758–2.682	0.271	2.023	1.131–3.619	0.018
(Quartile 2 vs. Quartile 4)	3.562	1.907–6.652	< 0.001	1.124	0.596–2.119	0.718	2.559	1.444–4.534	0.001
(Quartile 3 vs. Quartile 4)	2.417	1.278–4.569	0.007	0.806	0.417–1.557	0.521	1.798	1.008–3.204	0.047
Model 3									
BMI (kg/m^2^)	1.131	0.884–1.446	0.328	0.711	0.535–0.944	0.019	0.972	0.772–1.223	0.807
Waist circumference (cm)	0.979	0.770–1.244	0.860	0.724	0.550–0.954	0.022	0.893	0.713–1.118	0.323
HOMA-IR	1.220	0.742–2.007	0.433	0.995	0.586–1.691	0.985	1.068	0.671–1.700	0.780
Body fat mass (Kg)	1.040	0.817–1.323	0.751	0.775	0.591–1.016	0.065	0.970	0.773–1.217	0.791
Visceral fat (cm^2^)	1.674	1.302–2.153	< 0.001	0.767	0.584–1.007	0.057	1.210	0.955–1.532	0.115
Serum adipsin (ug/ml)	0.655	0.509–0.843	0.001	0.848	0.670–1.072	0.168	0.665	0.532–0.832	< 0.001
Serum adipsin (ug/ml)									
(Quartile 1 vs. Quartile 4)	1.935	0.976–3.836	0.059	1.528	0.799–2.923	0.200	2.267	1.236–4.158	0.008
(Quartile 2 vs. Quartile 4)	4.248	2.195–8.224	< 0.001	1.221	0.637–2.339	0.548	2.963	1.630–5.386	< 0.001
(Quartile 3 vs. Quartile 4)	2.742	1.411–5.328	0.003	0.809	0.413–1.584	0.537	1.910	1.049–3.475	0.034

^*^Asymptomatic carotid atherosclerosis is defined as increased CIMT or presence of atherosclerotic plaque

OR = odds ratio; CI = confidence interval; MI = body mass index; CIMT = Carotid intima-media thickness; BMI = body mass index; HOMA-IR = homeostasis model assessment of insulin resistance;

Model 1: adjusted for age, gender

Model 2: adjusted for model 1 + smoking, alcohol consumption, and physical activity

Model 3: adjusted for model 2 + SBP, glucose, total cholesterol, triglyceride, and HDL-c

## Discussion

Adipsin is recently identified as a novel adipokine that may modulate systemic energy metabolism and play a critical pathogenic role in obesity-associated metabolic diseases [21, 23, 29]. In the present study, we provide novel evidence that circulating adipsin concentrations were significantly reduced in subjects with asymptomatic carotid atherosclerosis and were inversely associated with increased CIMT in obese adults. However, serum adipsin was not significantly associated with atherosclerotic plaques. These findings indicate that circulating adipsin could be a potential marker in the development of CVD.

Adipsin is secreted mainly by adipose tissue and enhanced significantly during the progress of adipose tissue synthesis, which plays a vital role in obesity-related metabolic diseases, including type 2 diabetes and NAFLD [21, 30, 31]. Recently, adipsin has been identified as playing a pivotal role in preserving beta cells through controlling the complement pathway and generation of complement component C3a in diabetic mice and associates with protection from type 2 diabetes in humans [21, 23]. Some studies reported that β cell failure and poor glycemic control in Type 2 diabetes were inversely correlated to circulating adipsin levels [23, 32]. In addition, Liu et al. reported that serum adipsin concentrations were negatively associated with circulating cardiac markers [33]. It also has been reported that circulating adipsin levels were higher in overweight/obesity subjects and associated with increased cardiovascular risk in patients with polycystic ovary syndrome [34, 35]. Our study found that serum adipsin levels positively correlated with waist circumference and body fat, whereas another study reported that circulating adipsin levels were associated with subcutaneous fat rather than visceral adiposity [23]. Furthermore, Jun-Sing Wang and colleague reported that serum adipsin levels were negatively associated with insulin resistance in 320 subjects with various degrees of glucose intolerance, especially in subjects with a BMI ≥ 25 kg/m^2^ or prediabetes [24]. Consistently, our findings indicated that those in the highest quartile of serum adipsin levels had significantly lower levels of postprandial glucose and HOMA-IR. These findings suggest that adipsin may be a potential marker for metabolic syndrome.

Previous studies indicated that adipsin was associated with protection from type 2 diabetes and lower levels of metabolic risk in humans [21, 23]. In a cohort of 9771 healthy middle-aged men, higher plasma levels of adipsin was associated with an increased 10-year risk of ischemic stroke [36]. However, the role of serum adipsin levels in predicting incident CVD is still not conclusive. In particular, the relationship between circulating adipsin and subclinical atherosclerosis in human subjects remains largely unclear. Our present study provides novel evidence that circulating adipsin concentrations were significantly reduced in obese Chinese adults with asymptomatic carotid atherosclerosis and were inversely associated with increased CIMT. In addition, the relationship between circulating adipsin concentrations and CIMT may be modified by age. These findings suggest that serum levels of adipsin could be a potential marker in the development of CVD.

There are several potential reasons for circulating adipsin as a potential marker in the development of CVD in obese adults. First, serum adipsin level was negatively correlated with Type 2 diabetes [23] and other risk factors of CVD, including postprandial glucose and HOMA-IR. Thereby, it is no surprise that circulating adipsin concentrations could predict the development of CVD. Second, it can regulate glucose metabolism and vascular function. In this regard, circulating adipsin may regulate the complement replacement pathway to catalyze the production of the C3a and modulate vascular endothelial function (21, 37, 38). Therefore, circulating adipsin may be a potential marker for the risks of CVD in obese subjects. Prospective cohort studies are needed to confirm this finding and elucidate the potential underlying mechanisms.

This community-based, cross-sectional study provided an opportunity to determine the role of circulating adipsin in predicting the development of CVD and increased CIMT. There are certain limitations of our study. First, the population consisted of only obese adults with a relatively limited sample size in the present study. It is reported that adipsin has a correlation with adiposity and metabolic risk factors, which may confound the association of adipsin with metabolic diseases and the ability of adipsin to predict clinical outcomes. However, we found independent associations between circulating adipsin and outcomes even after adjusting for multiply potential confounders. Then we need further studies to determine the role of circulating adipsin in the development of CVD in the general population. Second, due to this study’s cross-sectional observational design, we could not determine whether there was a true causal relationship between circulating adipsin concentrations and the development of CVD. Therefore, further studies with long-term follow-up periods and larger sample sizes are warranted. Third, the study was limited to the Chinese population. Furthermore, the aim of our current study was defined as subclinical atherosclerosis rather than clinical cardiovascular events. Further studies need to determine the role of serum adipsin in predicting CVD events in different populations.

## Conclusions

In conclusion, these findings indicate that circulating adipsin concentrations are inversely associated with the risk of increased CIMT and asymptomatic carotid atherosclerosis in obese Chinese adults, suggesting that circulating adipsin concentrations may be a potential marker in the development of CVD and would be useful to improve strategies for CVD prevention.

## Data Availability

Data are available on request from the corresponding author.

## Abbreviations

CVD: Cardiovascular disease
CIMT: Carotid intima-media thickness
T2DM: Type 2 diabetes mellitus
MetS: Metabolic syndrome
BMI: Body mass index
BP: Blood pressure
HDL-c: High-density lipoprotein cholesterol
TG: Triglycerides
TC: Total cholesterol
LDL-c: Low-density lipoprotein cholesterol
HOMA-IR: Homeostasis model assessment of insulin resistance
GLM: General linear models
